# The Role of Substance P in the Regulation of Bone and Cartilage Metabolic Activity

**DOI:** 10.3389/fendo.2020.00077

**Published:** 2020-02-28

**Authors:** Fu-Xing-Zi Li, Feng Xu, Xiao Lin, Feng Wu, Jia-Yu Zhong, Yi Wang, Bei Guo, Ming-Hui Zheng, Su-Kang Shan, Ling-Qing Yuan

**Affiliations:** ^1^Department of Endocrinology and Metabolism, National Clinical Research Center for Metabolic Disease, Hunan Provincial Key Laboratory of Metabolic Bone Diseases, The Second Xiang-Ya Hospital, Central South University, Changsha, China; ^2^Department of Radiology, The Second Xiang-Ya Hospital, Central South University, Changsha, China; ^3^Department of Pathology, The Second Xiang-Ya Hospital, Central South University, Changsha, China

**Keywords:** substance P, Nk-R1 (neurokinin-receptor 1), osteoblasts, osteoclasts, osteoporosis, fracture healing, osteoarthritis

## Abstract

Substance P (SP) is a neuropeptide that is released from sensory nerve endings and is widely present in nerve fibers. It acts on bones and related tissues by binding to receptors, thereby regulating bone metabolism, cartilage metabolism, and fracture healing. SP has attracted widespread attention as a signaling substance that can be recognized by both the immune system and the nervous system. Previous studies have shown that bone and chondrocytes can synthesize and secrete sensory neuropeptides and express their receptors, and can promote proliferation, differentiation, apoptosis, matrix synthesis, and the degradation of target cells through autocrine/paracrine modes. In this paper, we review the research progress made in this field in recent years in order to provide a reference for further understanding the regulatory mechanism of bone and cartilage physiology and pathological metabolism.

## Introduction

Bone is a complex and dynamic tissue with a mineralized extracellular matrix and the ability to adapt to its functional demands and repair itself. Bone is abundantly innervated by small diameter sensory nerves in the periosteum, bone marrow, and vascular canals (1, 2). There is increasing evidence that the sensory nervous system is one of the key factors in bone cell differentiation, bone metabolism, and bone remodeling. Neuropeptide plays an important role in the balance between bone formation and bone resorption, and its role in bone repair and reconstruction has gradually become a hot topic. It has been reported that SP is closely related to bone metabolism. SP was accidentally isolated from the brain and intestine extracts of horses by Euler and Gaddum (3). SP is an 11-amino acid peptide that is widely distributed in the peripheral and central nervous system (1, 4). The amino acid sequence is as follows: H-Arg-Pro-Lys-Pro-Gln-Gln-Phe-Ple-Gly-Leu-Met-NH2 (5). SP belongs to the tachykinin neuropeptide (TK) family and is the major neuropeptide synthesized from the Tac1 (pre-protachykinin-A) gene. TK mediates its biological effects through three different NK (neurokinin 1, 2, 3) receptors. Among these, NK1 (also known as the tachykinin 1 receptor, TACR1) (6) has the highest affinity for SP and is the main receptor of SP (7–9). SP is widely distributed in the central and peripheral nervous systems as well as in various tissues and organs. SP-like nerve fibers are distributed in various bone tissues of the human body, including long bones, joints and teeth, and most of the metabolically active parts such as the periosteum and epiphyseal growth plate are distributed, and the cortex and bone marrow are relatively small at the backbone (4, 10). SP is also found in chondrocytes, subchondral bone, and cartilage membranes (11). SP is known to be involved in many physiological and pathophysiological processes including vasodilation, extravasation, smooth muscle contraction (12), pain transmission (13), neurogenic inflammation (14), angiogenesis and bone turnover (15, 16). Cartilage, which contains no blood vessels, nerves, or lymphatics, has long been considered inert tissue in the body. However, an increasing number of studies have shown that cartilage is also regulated by sensory nerves, and sensory neuropeptides can be involved in the regulation of cartilage physiological and pathological metabolism by affecting the proliferation, differentiation, and secretion of chondrocytes (17–20). Although SP and its receptors are widely distributed in the locomotor system, its effects on bone and cartilage metabolism are not well-understood. In the present review, we focus on the effects of SP on bone and cartilage metabolism in some physiological and pathological states. The objective of this article is to review the modulatory effects of SP on the skeletal system and to afford a comprehensive understanding of SP and bone/cartilage metabolism.

## SP and Its Receptor in Bone Diseases

### Bone Remodeling

**Figure 1:**
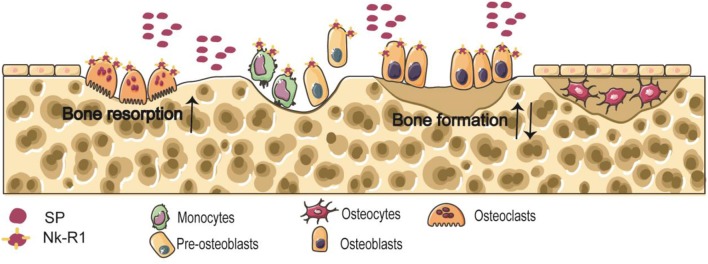
Schema of the effects of released SP on the osteoclasts and osteoblasts.

The balance between osteoclastic bone resorption and osteogenic bone formation processes in bone metabolism is the key to maintaining normal bone mass (21). Osteoclasts and osteoblasts are derived from bone marrow macrophages and bone marrow stromal cells (BMSCs), respectively (22). Neuropeptides regulate the functions of osteoclasts and osteoblasts by binding to receptors, and participate in bone growth, repair and reconstruction. Nk-R1 is expressed by osteoblasts and osteoclast precursors (22) (Figure 1).

#### SP and Osteoblasts

Osteoblastic progenitors are derived from mesenchymal stem cells (MSCs) (23) and require Runx2 (also known as Cbfa1, runt-related transcription factor2) transcription factors to develop into a mature osteoblastic lineage. They are characterized by an osteoblastic morphology, accompanied by an increase in alkaline phosphatase (ALP) activity and the production of type I collagen and osteocalcin (24). Osteoblasts not only participate in bone formation, but also regulate osteoclast differentiation by secreting macrophage colony stimulating factor (m-csf), receptor activator of nuclear factor-κB ligand (RANKL), and osteopectin (OPG) receptor activators (25). However, the effect of SP on bone formation remains unclear. Interestingly, Goto et al. (26) added SP to rat calvarial osteoblasts to enhance mineralized nodular formation, and two different NK1 antagonists—spantide and FK888—inhibited the expression of Runx2, OCN and type I collagen, which suggested that blocking the SP receptor attenuates osteogenic differentiation. Wang et al. (22) showed that a low SP concentration can promote the proliferation of BMSCs, while a high SP concentration can promote the mineralization of bone marrow stromal cell. SP can improve the bone formation activity of late osteoblasts by acting on Nk-R1 (26). Shih et al. (27) found that *in vitro* culture of bone marrow cells, the number, and size of bone marrow cell colonies increased with SP concentration, indicating that SP could stimulate bone formation. Paradoxically, SP slightly stimulated thymidine incorporation and strongly inhibited calcium accumulation in bone noduli, which was associated with a small decrease in ALP activity (28). SP promoted osteoblast proliferation and inhibited differentiation and mineralization in rats with spinal cord injury through the RANKL/OPG system (29). Although the results of such studies are inconsistent, it is certain that the formation of osteoblasts is influenced by SP. Further research is needed to clarify how SP regulates bone differentiation, what mechanisms are involved in bone formation, and which neurokinin receptors SP activates in osteoblasts and osteocytes.

#### SP and Osteoclasts

Mature osteoclasts are terminally differentiated, multinucleated cells, formed by the fusion of hematopoietic stem cells through the myelomonocytic precursor cells/macrophage lineage (30). Early stages of osteoclast differentiation are initiated by the binding of m-csf to its receptor c-fos, which, in turn, induces the expression of receptor activator of nuclear factor-κB (RANK), a membrane protein expressed by preosteoclasts. The interaction between RANK and RANKL, which is expressed by cells of the osteoblast lineage, is the major trigger of osteoclast differentiation and activation (31). Tanja et al. (32) found that a lack of SP reduced the bone resorption rate and reduced the numbers of bone marrow precursor cells (BMMs) and multinucleated osteoclasts and reduced cathepsin K activity in tachykinin (Tac)1^−/−^ BMMs/osteoclast cultures. Takaaki et al. (33) proposed a new mechanism for inducing osteoclast formation by stimulating synovial cells with SP. They showed that SP released from peripheral sensory nerve endings is one of the risk factors for the development of arthritis. It induces synovial hyperplasia and hypertrophy, up-regulates RANKL expression and down-regulates the expression of OPG in synovial fibroblastic cells, which results in osteoclastogenesis. SP-like nerve fibers are closely related to the reconstruction of orthodontic periodontal tissue. In patients with severe root resorption of orthodontic teeth, SP can increase the production of pro-inflammatory cytokines and osteoclast formation in pulp fibroblasts (34). Bone loss in capsaicin-treated animals was associated with a decrease in the rate of bone formation and an increase in the number of osteoclasts and the function of osteoclasts (15). Given the above evidence, it is paradoxical that excessive release of SP leads to increased bone absorption, and that extensive reduction of SP in bones also leads to osteoporosis.

SP may maintain a balance between bone resorption and bone formation and mediate bone resorption depending on whether its level is greater or less than a specific range, suggesting that different amounts of SP affect bone metabolism through different mechanisms. Caye-Thomasen et al. found that in the early stages of acute otitis media, it was SP release that caused increased bone tissue absorption in the middle ear (35). NF-κB is an essential transcription factor for osteoclastogenesis (36). At the same time SP upregulates osteoclastogenesis by activating the NF-κB in osteoclast precursors (36), similar to the effects of RANKL. Wang et al. (22) also observed that SP up-regulated osteoclastogenesis in BMMs and RAW 264.7 cells and increased bone resorption in BMMs. SP appears to enhance RANKL-induced osteoclastogenesis and bone resorption in the same manner as tumor necrosis factor-a (TNF-a) (37). The addition of RANKL to osteoclast cultures induces the release of Ca^2+^ from intracellular storage, leading to an instantaneous increase in intracellular free Ca^2+^, which accelerates nuclear translocation of NF-κB (38, 39). Similarly, treatment with SP increased cytoplasmic Ca^2+^ levels in rabbit osteoclasts due to an influx of extracellular Ca^2+^ (40). Mori et al. (40) demonstrated that the addition of SP to cultured rabbit osteoclasts resulted in an acute rise in intracellular calcium concentration, which was eliminated by SP receptor antagonists. Intracellular Ca^2+^ mobilization may be a common signaling pathway for RANKL and SP activation of NF-κB in macrophages and osteoclasts. Some scholars have observed that cutting off the sympathetic nerve can promote an increase in peripheral SP and bone absorption (41, 42). The reason may be that after the sympathetic nerve is cut off, the intake of nerve growth factor in the bone tissues it innervates will be reduced, so the sensory nerve will increase the intake of nerve growth factor, which will further promote the synthesis and peripheral release of SP in the sensory neurons. These findings clearly indicate the effects of SP in accelerating osteoclastic bone resorption.

Osteocytes are the main cell in mature bone tissue, equivalent to human adulthood. Osteoclasts/bone-resorbing cells are multinucleated cells formed from differentiated monocytes/macrophages. Osteoblasts/bone-forming cells, derived from pre-osteoblast cells, are the main functional cells of bone formation and responsible for the synthesis, secretion, and mineralization of bone matrix. Nk-R1 has been reported to exist on monocytes, pre-osteoblast cells, osteoblasts, and osteoclasts. Some findings clearly indicate the accelerated effect of SP on osteoclastic bone resorption. And the effect of SP on bone formation is still unclear.

### Osteoporosis

Osteoporosis is a critical risk factor for fragility fractures, causing substantial morbidity, and mortality, especially in postmenopausal women and the elderly (43). A large number of neuropeptides regulating bone metabolism may represent a regulatory pathway for the pathogenesis of osteoporosis. Liu et al. (44) reported that after constructing a model of osteoporosis in adult female rats, ovariectomy (OVX) reduced SP in the bone. Liu et al. (45) indicated that epimedium treatment reduced the effects of osteoporosis through a brain/spinal cord/bone axis by increasing bone SP. Interestingly, spinal cord injury (SCI) in experimental rats resulted in an osteoporotic phenotype in the proximal tibia, due to enhanced osteoclast uptake, which was associated with a substantial increase in SP immunoreactive nerve fibers, consistent with *in vitro* observations of SP enhancing osteoclast activity (46). BMD and bone microstructure were significantly reduced at 3 weeks after mechanical stimulation of SCI. The mechanism of OP following SCI is still unclear. Some possible explanations for the pathogenesis of OP after SCI. Firstly, metabolic function changes, such as impaired renal function, hyperlipidemia, and insulin resistance, which may be related to the pathogenesis of OP after SCI. The second is the reduced mechanical loading, the third explanation responsible for this process in the nerve injury itself (46). Chen et al. (47) reported that gelatin microspheres containing different concentrations of SP promoted osteogenesis after 3 months in a rabbit osteoporotic bone defective model. SP increased the amount of trabecular bone and reduced trabecular bone separation. Histological analysis showed that the gelatin microspheres containing SP effectively promoted osteogenesis, regardless of the concentration. Zheng et al. (48) revealed that SP expression decreased in the bone of OVX mice following application of L-703606 (Nk-R1-specific antagonist), and bone loss and the degeneration of bone microstructure in OVX mice was accelerated. Biomechanical analysis showed that blockade of SP signaling can reduce the maximum stress and maximum load of L3 vertebrae and tibiae. In mice treated with L-703606, there was an increase in the number of osteoclasts, a decrease in the number of osteoblasts and an increase in the osteoid volume in the secondary spongiosa, thereby inhibiting the recruitment of BMSCs to the bone reconstruction site. The OPG/RANKL ratio in the bone of mice treated with L-703606 was also significantly decreased. Kingery et al. (49) suggested that a significant reduction in SP could lead to osteoporosis after sciatic neurectomy. Based on the evidence above, it is possible that the pathogenesis of osteoporosis is associated with the regulation of SP. SP signaling certainly plays an important role in the maintenance of bone mass.

### Fractures

SP is considered to be a regulator of angiogenesis that is important for bone repair and remodeling. Ding et al. (50) built a model in which femoral shaft fracture was created 3 weeks after OVX exposure. The fracture healing ability of young mice with OVX-induced bone loss was significantly worse than that in control mice, and SP in the fracture site was significantly decreased at all time points. It was also found that angiogenesis was impaired in OVX mice. The results suggest that neural regulation may play a role in osteoporotic fracture healing and that SP plays an important role during fracture healing, particularly in the early stages. These data contribute to the evidence that SP may play an important role in osteoporotic fracture healing. Tanja et al. (51) used wild type mice and SP-deficient mice (Tac1^−/−^) to establish a fracture healing model to study the effect of SP loss on the process of fracture healing. At day 13 post fracture, they observed a decrease in the area covered by hypertrophic chondrocytes in Tac1^−/−^ mice, indicating that SP deficiency can delay the terminal differentiation of hypertrophic chondrocytes. This research suggested that SP is essential in the process of cartilage ossification during fracture healing. Furthermore, absence of SP reduces pain sensitivity and the mechanical stability of the bone after fracture in general. Guo et al. (52) showed that the NK-R1 antagonist LY303870 partially reversed the vascular and traumatic sequelae of tibial fractures in rats, demonstrating the important role of SP in fractures. Although the effects of SP on osteoblasts and osteoclasts remain controversial, SP plays a critical role in maintaining the balance between bone resorption and bone formation by regulating osteoblasts and osteoclasts during fracture healing (53, 54). Based on the correlation between neuropeptides and fracture healing, the authors hypothesized that SP is secreted into the surrounding bone tissue in a certain way. The amount of this neuropeptide changes during fracture healing, and SP binds to receptors on the cell membrane, activating intracellular signaling pathways, which in turn affect fracture healing and later bone remodeling.

### Chronic Inflammation

SP is a neuroinflammatory mediator produced by sensory nerve fibers and local inflammatory cells, such as macrophages, lymphocytes, and dendritic cells (53, 55). SP plays an important role in the skeletal degeneration and damage induced by chronic inflammation (4). Abnormal expression of SP and Nk-R1 in inflammatory diseases provides evidence for SP's involvement in the inflammatory response. Knocking out the Nk-R1 gene and applying an Nk-R1 antagonist in animal models of inflammatory diseases have significant anti-inflammatory effects. SP plays an important role in the development of arthritis as evidenced by a positive correlation between the size and severity of joint destructive changes (56). Rheumatoid arthritis (RA) is a systemic autoimmune disease associated with chronic inflammation of connective tissue. Recent evidence suggests that SP and its receptors are involved in joint inflammation and are involved in the pathophysiology of RA (57). SP levels and Nk-R1 expression were increased in the synovial fluid obtained from RA patients (58). SP stimulated RA synovial cells to release prostaglandin E2 and collagenase, which in turn increased the proliferation of RA synovial cells (59). This suggests that SP plays a role in the development of cartilage destruction and bone damage in arthritis. Experiments with chronic arthritis in rats have shown that sustained inflammatory stimulation can increase SP release in the spinal cord horn (60). Chronic inflammation is a common symptom in OA (osteoarthritis) and RA. A study by Barbara et al. (61) found that serum SP concentration in patients with OA and RA was positively correlated with the intensity of chronic pain.

## Physiological and Pathological Effects of SP on Cartilage Metabolism

### Physiological and Pathological Effects

SP seems to be extremely important for cartilage health because it participates in mechanical transduction through Nk-R1 (62–64). Millward-Sadler et al. (65) demonstrated that adult human articular chondrocytes expressed endogenous pre-protachykinin (PPT, an SP precursor) mRNA, SP, and the corresponding Nk-R1 *in vivo* and *in vitro*. The addition of 1 μmol/L SP to cultured chondrocytes or 0.33 Hz of mechanical stimulation caused hyperpolarization of the cell membrane, suggesting that SP is involved in the mechanical transmission process in chondrocytes. Blockade of SP signaling by a chemical antagonist of Nk-R1 inhibited chondrocyte responses to mechanical stimulation. To sum up, SP secreted by human articular chondrocytes can mediate chondrocyte mechanotransduction via Nk-R1 in an autocrine and/or paracrine manner. Karaha et al. (66) revealed that there was moderate SP expression in articular chondrocytes in a low-exercise group, but SP expression in the cartilage matrix was low or absent. However, SP expression in articular chondrocytes, cartilage matrix, and synovial membrane cells was significantly higher in a high-exercise group, suggesting that SP plays a role in regulating the physiological microenvironment of the cartilage, metabolism, and joint function.

### Pathological Effects—Fracture Healing

Opolka et al. (67) conducted studies on costal chondrocytes of 3-week-old mice *in vitro* and found that SP significantly promoted the gene expression of type I, IX, X collagen, and mmp-13, which are closely associated with the terminal differentiation of chondrocytes. In addition, SP also promoted chondrocyte proliferation in a dose-dependent manner. It was also found that the NK1 antagonist L733060 could exert dose-dependent inhibition on the proliferation of chondrocytes, indicating that endogenous synthesis and secretion of chondrocyte SP could also regulate proliferation through autocrine and paracrine effects. Tanja et al. (51) found at the stage of cartilage nodules formation in fracture healing mice, and a large number of chondrocytes were observed to accumulate. Notably, the fracture chondrocytes expressed high levels of SP and its receptor NK-1 (68), and their expression level was regulated by nerve growth factor and inflammatory factor (11, 51, 69, 70). Future therapeutic targets may involve blocking this particular receptor.

### Pathological Effects—Osteoarthritis

Osteoarthritis, also known as degenerative joint disease, is characterized by synovial inflammation, cartilage destruction, and subchondral bone sclerosis associated with aging. Joint replacement is still the only treatment for patients with advanced osteoarthrosis. Recent studies have shown that SP plays an important therapeutic role in the process of OA cartilage degeneration. Higher concentrations of SP were found in the synovial fluid of OA patients, indicating the catabolic effect of SP on articular cartilage (71, 72). Increased substance P levels have been reported in synovial fluid and cerebrospinal fluid obtained from OA patients (73) and immunohistochemistry has demonstrated an increase in SP-immunoreactive nerve fibers in patients with OA (74), also indicating the catabolic effect of SP on articular cartilage (75). Although cartilage is not innervated, increased release of SP in sensory nerve endings during synovial inflammation may affect chondrocyte function. Alternatively, the release of SP by chondrocytes through mechanical stimulation or by other means may affect the activity of various cell types in joints and periarticular tissues (including macrophages, bone cells, and pain fibers), as well as the structural changes associated with OA. Suri et al. (76) found that during the development of OA, new blood vessels could break through the junction of osteochondral cartilage, and sensory nerve fibers could also grow into the diseased cartilage tissue along with blood vessels. At this time, neurogenic SP could act on NK1 in the cartilage cell membrane in a paracrine manner, thus accelerating cartilage degeneration. In animal models and human studies of OA, SP, and NK receptors have been linked to joint pain, inflammation, and injury (77, 78).

Although studies have shown that SP receptor antagonists can help reduce arthritis pain and swelling (79), blocking SP can reduce pain but increase the rate of changes in OA arthritis (80). Therefore, in the treatment of OA, SP may have a dual opposite effect (OA treatment through its anti-inflammatory effect and endogenous stem cell recruitment and pain relief by lowering the pain threshold). A pre-clinical study with a hydrogel implant in a rat knee model revealed that this contradictory potential of SP for therapeutic application in OA can be resolved by the adjustment of the SP dose, the continuity of SP release, and the use of an adequate conjugate to modify the properties of SP. In the same study, SAP conjugates (SP with self-assembled peptide) were used to treat OA. The treatment of OA with SAP-SP significantly improved cartilage regeneration by recruiting MSCs. SAP-SP can prevent apoptosis by secreting anti-inflammatory cytokines, increasing the amount of extracellular matrix involved in chondrogenesis, promoting chondrogenesis and differentiation, and reducing inflammation in OA (78). These studies suggest that SP exhibits anti-inflammatory and regenerative properties through the recruitment of MSCs.

## Conclusion

Previous studies have confirmed that SP and Nk-R1 are widely present in bone and cartilage tissue and actively participate in bone and cartilage metabolism. Sensory nerve endings can release SP, and SP-positive nerve fibers are distributed in bone and cartilage tissue. SP binds to Nk-R1 to initiate a signal transduction pathway and regulates pathophysiological processes in bone and cartilage tissue.

Due to the current lack of detailed knowledge about the effects of SP on bone, we are still unable to explain the pathophysiology of the most common bone diseases. Although previous studies have shown that SP is involved in bone metabolism, especially in bone resorption, the effect of SP on osteoblast formation is not fully understood. It will be a challenge to elucidate the relationship between bone metabolism and neural regulation. As a substance that can be synthesized by the human body, SP can produce a rapid and efficient response at very low levels. It remains to be determined whether it could be a potential therapeutic agent in osteoporosis and fracture repair. The exact mechanism by which SP is involved in the pathophysiology of bone and related tissues, the interaction of SP with other neuropeptides, cytokines and hormones, and the potential role of SP or Nk-R1 antagonists as effective preventive and therapeutic agents need to be fully determined.

SP plays an important regulatory role in the mechanical response of cartilage, fracture healing, and the pathological degeneration of cartilage by promoting chondrocyte proliferation, adhesion, and secretion and accelerating chondrocyte terminal differentiation. Although cartilage metabolism involves multiple pathways, and SP is not the main pathway that plays a regulatory role, it has been proposed that through SP, NK-R1 antagonists may be promising for the treatment of OA in the future.

Future research should explore areas related to the changes in SP and its receptors in cell proliferation and differentiation, cell signal transduction pathways, and protein and gene expression levels after SP treatment of animals/cells *in vivo* or *in vitro*. Research on SP, the NK1 receptor and the Nk-R1 antagonist will be useful for exploring the mechanism of action of drugs, developing new drugs, and finding new treatments for bone diseases.

## Author Contributions

L-QY conceived and designed the manuscript. F-X-ZL, FX, XL, FW, J-YZ, YW, BG, M-HZ, and S-KS analyzed the data. L-QY and F-X-ZL wrote the paper.

### Conflict of Interest

The authors declare that the research was conducted in the absence of any commercial or financial relationships that could be construed as a potential conflict of interest.
